# Gender Differences in Cooperation: Experimental Evidence on High School Students

**DOI:** 10.1371/journal.pone.0083700

**Published:** 2013-12-18

**Authors:** J. Alberto Molina, J. Ignacio Giménez-Nadal, José A. Cuesta, Carlos Gracia-Lazaro, Yamir Moreno, Angel Sanchez

**Affiliations:** 1 Departamento de Análisis Económico, Universidad de Zaragoza, Zaragoza, Spain; 2 Institute for the Study of Labor-IZA, Bonn, Germany; 3 Instituto de Biocomputación y Física de Sistemas Complejos, Universidad de Zaragoza, Zaragoza, Spain; 4 Grupo Interdisciplinar de Sistemas Complejos, Departamento de Matemáticas, Universidad Carlos III de Madrid, Leganés, Spain; 5 Complex Networks and Systems Lagrange Laboratory, Institute for Scientific Interchange, Torino, Italy; University of Maribor, Slovenia

## Abstract

The emergence of cooperation among unrelated human subjects is a long-standing conundrum that has been amply studied both theoretically and experimentally. Within the question, a less explored issue relates to the gender dependence of cooperation, which can be traced back to Darwin, who stated that "women are less selfish but men are more competitive". Indeed, gender has been shown to be relevant in several game theoretical paradigms of social cooperativeness, including prisoner's dilemma, snowdrift and ultimatum/dictator games, but there is no consensus as to which gender is more cooperative. We here contribute to this literature by analyzing the role of gender in a repeated Prisoners' Dilemma played by Spanish high-school students in both a square lattice and a heterogeneous network. While the experiment was conducted to shed light on the influence of networks on the emergence of cooperation, we benefit from the availability of a large dataset of more 1200 participants. We applied different standard econometric techniques to this dataset, including Ordinary Least Squares and Linear Probability models including random effects. All our analyses indicate that being male is negatively associated with the level of cooperation, this association being statistically significant at standard levels. We also obtain a gender difference in the level of cooperation when we control for the unobserved heterogeneity of individuals, which indicates that the gender gap in cooperation favoring female students is present after netting out this effect from other socio-demographics factors not controlled for in the experiment, and from gender differences in risk, social and competitive preferences.

## Introduction

The question about whether or not cooperation of individuals varies systematically with the sex of the decision maker has generated considerable debate. If such difference is present, it will probably affect the modeling of economic outcomes such as household bargaining or intergenerational transmissions, among others. In the household bargaining framework [1,2], model the distribution of resources within couples (e.g., income, consumption) as a solution to a cooperative game, usually a Nash bargaining point, in which the threat point is divorce. More recently [3], developed a super-game in which the spouses play a non-cooperative Stackelberg game where the leader first decides the contributions to a certain quantity of provision of family good, and thereby sets restrictions for the follower. If women are more cooperative than men, this would affect the modeling of the bargaining process within couples. In the intergenerational transfers setting, if mothers are more cooperative than fathers, this could help explain why mothers devote more time to childcare activities than their male counterparts [4-14]. Thus, the study of gender differences in cooperative behavior is important at both the theoretical and empirical level. 

Under this framework, we provide empirical evidence on gender differences in cooperation between individuals by developing a repeated prisoner’s dilemma experiment. Prior research on the prisoner’s dilemma, social dilemmas, and public goods provision has found mixed results from a gender perspective. Psychological studies analyzing gender differences in the prisoner’s dilemma setting showed that men cooperate significantly more than women [15-17] on the contrary, other studies showed that women are more cooperative than men [18,19], while others found no significant differences in cooperation [20,21]. From an evolutionary biology perspective [22], found that women are more cooperative than men in Prisoner’s Dilemma games, but not in the Snowdrift’s game. In the field of economic experiments, evidence has been in favor of a gender gap in cooperation favoring women [23]. showed that women are significantly more cooperative than men in prisoner’s dilemma games [24], found the same gender difference in the first round of the game, the difference subsequently disappearing over time. Thus, previous evidence about gender differences in cooperation is mixed, although it seems that evidence in the field of economics points toward women being more cooperative than men from a social dilemma perspective.

We contribute to the literature by analyzing gender differences in cooperation, specifically for high school students, by developing an experiment with 1,229 volunteers from final-year high-school students (17-18 years old) of 42 high schools located in the Region of Aragón. The experiment includes 2 phases of a multiplayer prisoner’s dilemma, where in the first phase players’ partners are the same for all the 51 rounds while in the second phase of the experiment players’ partners change in all the 59 rounds. The fact that we have a large sample of individuals with several observations per individual allows us to disentangle the effect of gender from the effect of other factors that may bias the results, such as that of the unobserved heterogeneity of individuals. Standard econometric techniques used in the field of economics (i.e., Ordinary Least Squares and Random Effects models) are applied to analyze gender differences in the level of cooperation among high school students. 

We find that, in all our specifications and phases of the experiment, being male is negatively associated with the level of cooperation, with this association being statistically significant at standard levels. In particular, male students have a probability between 4 and 8 percentage points lower of cooperating compared to male students. We also obtain a gender difference in the level of cooperation when we control for the unobserved heterogeneity of individuals, which indicates that the gender gap in cooperation favoring female students is present after netting out this effect from other socio-demographics factors not controlled for in the experiment, and from gender differences in risk, social and competitive preferences (see 25 for a review). Thus, our results point toward a gender difference in the level of cooperation that may be attributed to a genetic factor. The fact that we obtain similar results when we use alternative subsamples and econometric techniques indicates that our results are good enough to draw valid conclusions.

The rest of the paper is organized as follows. Section 2 presents the experiment. Section 3 describes the empirical strategy. Section 4 presents our main results, and Section 5 sets out our main conclusions.

## Methods

### Ethics statement

All participants in the experiments reported in this manuscript signed an informed consent to participate. Besides, their anonymity was always preserved (in agreement with the Spanish Law for Personal Data Protection) by assigning them randomly a username which would identify them in the system. No association was ever made between their real names and the results. As it is standard in socio-economic experiments, no ethic concerns are involved other than preserving the anonymity of participants. This procedure was checked and approved by the Viceprovost of Research of Universidad de Zaragoza, the institution hosting the experiment.

### The experiment: Prisoner's Dilemma

The experiment was carried out with 1,229 volunteers selected from final-year high-school students (17-18 years old) of 42 different High Schools located in the Region of Aragón (Spain). In order to satisfy ethical procedures, all personal data about the participants were anonymized and treated as confidential. The Ethical Committee of the University of Zaragoza approved all procedures. 34 High Schools were selected in the province of Zaragoza, 5 in the province of Huesca, and 3 in the province of Teruel. For the recruitment of the students, the coordinators of “Ciencia Viva” ("Living Science"), a program of the regional government that supports the dissemination of Science among public high schools in Aragón, were contacted. Many of the private schools of Zaragoza City were also contacted, offering them the possibility of taking part in the experiment. In all cases, the program was referred to as "a social experiment" and no-one (including the high-school teachers in charge of the coordination) knew in advance what the experiment was about. The final sample of volunteers comprises 541 males and 688 females representing 44.02% and 55.98% of the total number of players, respectively.

Out of the 1,229 students, 625 played the game as nodes on a square lattice (274 males and 351 females, maintaining the male-female ratio), and 604 on a heterogeneous network. In both topologies, players played a prisoner’s dilemma with all their neighbors, restricted to choosing the same action for every opponent (see below). In the first topology, every player had k=4 neighbors, while in the second network the connectivity varied between 2 and 16. In the first phase of the experiment, the network was static, i.e., every player interacted with same partners throughout the duration of that part. In a subsequent phase, neighbors were randomly assigned in each round, taking care in the heterogeneous case of keeping the number of partners of every player constant. All the students played via a web interface, specifically created for the experiment, accessible through the computers available in the computer rooms of their respective schools. At least one teacher supervised the experiment in each computer room (which at most had a maximum capacity of 20 students), preventing any interaction among the students. To further guarantee that potential interactions among students seated next to each other in the class do not influence the results of the experiment, the assignment of players to the different topologies was completely random. Hence, the odds of having two participants geographically close (i.e., of the same school and seated next to each other) who were also neighbors in the virtual topology was quite small. Additionally, the colors used to code the two available actions of the game were also randomly selected for each player, thus decreasing the likelihood that neighbors would influence each other.

All participants went through a tutorial on the screen, including questions to check their understanding of the game. When every-one had gone through the tutorial, the experiment began, lasting for approximately an hour. At the end of the experiments volunteers were presented a small questionnaire to fill in. Immediately after, all participants received their earnings and their attendance fee, with total earnings ranging from 2.49 to 40.48 €. The experiment started on December 20, 2011 at 10:00 CET. The steps followed during the development of the experiment were (see tutorial for players in “S1. The Experiment” in Material S1 for a full description of the tutorial for players; see Figures S1, S2 and S3 in Material S1 for snapshots of the web interface):

1. Administrators opened the registration process.
2. Players (students) registered from their computers.
3. Once all students had registered, teachers informed the administrators via their screen.
4. As soon as the required number of participants had registered (this took around 20 minutes), administrators blocked further registrations and initiated the reading of the tutorial.
5. Students and teachers read the tutorial.
6. Teachers informed (also via their screens) administrators that the reading was completed.
7. Phase 1 of the experiment began, which lasted 59 rounds.
8. Students played according to some predefined times (a maximum of 20 seconds per round to choose an action). During these steps, teachers controlled for any potential problem using a chat channel that connected them to the administrators. If one student did not play within the 20 seconds allowed for each action, our software played automatically for her (see below). The administrators were able to identify who was not playing, and to contact the teachers if the situation persisted. However, the experiment went smoothly and no feedback to the teachers for mis-behavior was needed.
9. Phase 1 of the experiment ended and a brief tutorial on the second phase was shown.
10. Once teachers and students had read the tutorial, the administrators were notified.
11. Administrators began phase 2 of the experiment, which lasted 51 rounds.
12. Students played as in the previous phase.
13. Once phase 2 of the experiment ended, players were given a short questionnaire to fill in.
14. All participants collected their earnings and were given their show-up fee.

The experiment ended at 12:30 CET. The experiment did not have a fixed number of rounds for each phase, which explains why the 2 phases have a different number of rounds. We implemented a repeated Prisoner’s Dilemma where the game ended at any point between rounds 40 and 60 with equal probability.

### The Game: The Repeated Prisoner’s Dilemma

In each round, each participant is placed in a node of a virtual network, where participant “i” was linked to “j” (j=2, 3,..., 16) people (whom we shall refer to as neighbors), and where the actual number of neighbors was shown to each participant. Participant “i’s” neighbors were connected to other people, not necessarily the same ones as participant “i”. Participants did not know who their neighbors were.

**Figure 1:**
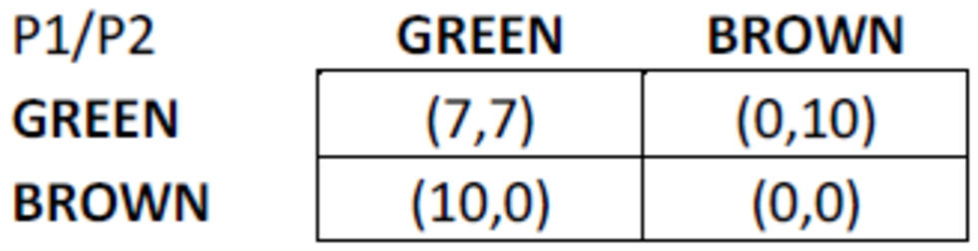
Earning structure of the Game. [*Note*: units are ECUs that were later converted to €.].

About the decision to be made in each round, each of the participants had to choose a color: GREEN or BROWN (each participant sees the actual colors chosen for them, for clarity, we henceforth refer to green and brown). To choose a color, participants simply had to click a button appearing on the screen. Each time the participants chose a color, they earned an amount of money that depended on their own and their “j” neighbors’ choices. The earnings of each round were given in a monetary unit called ECU. When the experiment ended, an exchange rate from ECUs to Euros was established as a function of the earnings of the participants and the budget available for the experiment (10 500 €). If participant “i” chose GREEN and her neighbor also chose GREEN, each participant received 7 ECUs. If participant “i” chose GREEN and her neighbor chose BROWN, participant “i” received 0 ECUs, and her neighbor received 10 ECUs. If participant “i” chose BROWN and her neighbor also chose BROWN, each received 0 ECUs. If participant “i” chose BROWN and her neighbor chose GREEN, participant “i” received 10 ECUs and her neighbor received 0 ECUs. These rules were the same for all participants. See Figure 1 for a representation of the game and earnings.

According to the structure of the game, participant “i” and each of her neighbors will globally earn more if both choose GREEN (7 ECUs participant “i”/7 ECUs her neighbors). However, participant “i” will earn more if he/she chooses BROWN and her neighbor chooses GREEN (10 ECUs participant “i”/0 ECUs her neighbor), while if both choose BROWN, participant “i” and her neighbor will each earn less (0 ECUs participant “i”/0 ECUs her neighbor) than if they both chose GREEN. The earnings of each pair of strategies follow the structure of a Prisoner’s Dilemma game that represents a situation in which two individuals may defect (e.g., BROWN), even if it appears that it is in their best interests to cooperate (e.g. GREEN): pursuing individual reward logically leads both players to defect, but they would get a better reward if they both cooperated. 

### Empirical Evidence

Table 1 shows means and standard deviations for our variable of interest (cooperation or defection) and several demographic and game characteristics. We have pooled all the rounds, networks and phases together to obtain average values of our variables of interest. Given that we use information on the previous round in our estimations and thus we will exclude the first round of the game, we show summary statistics excluding information from the first round of each experiment and control phase. We observe that the variable *Average cooperation* has a mean value of 0.341, indicating that individuals chose to cooperate in 34% of the rounds. Previous literature in experimental economics using prisoner’s dilemma situations have found “anomalous” cooperative behavior with 40% to 60% contribution rates in spite that defection in every game is the unique dominant-strategy Nash Equilibrium [26,27], which can be explained by the “sequential equilibrium reputation hypothesis” [28]. According to this hypothesis, reputation effects due to informational asymmetries can generate cooperative behavior in repeated versions of the classic prisoner’s dilemma as players may believe that there is a small chance that their opponent may be altruistic. Then it could be in each player's best interest to pretend, at least for some time, to be an altruistic player in order to build a reputation for cooperation, until the game eventually unravels to mutual defection.

**Table 1:** Sum stats for personal and game characteristics.

	**(1)**	**(2)**
**Variables**	**Mean**	**Standard deviation**
*RoundCharacteristics*		
***Cooperation***	0.341	(0.474)
***Earnings***	10.536	(9.621)
***Number of neighbors***	3.574	(1.504)
***Payoff in previous round***	10.647	(9.671)
***Mean payoff of neighbors in previous round***	12.745	(7.712)
*DemographicCharacteristics*		
***Male***	0.440	(0.496)
***Number of siblings***	1.117	(0.869)
***Following Humanities***	0.265	(0.441)
***Attending to a private school***	0.071	(0.256)
***Attending to a Semi-private school***	0.277	(0.448)
***Attending to an urban school***	0.746	(0.435)
*N Observations*	131,503	

Note: Sample consists of final-year high school students from Aragon (Spain). *Cooperation* is a dummy variable that takes value 1 if individual “i” in network “j” at round “t” decided to cooperate, and takes value 0 if he/she decided to defect. *Earnings* measures the payoff received by the reference player in each round. *Payoff* *in* *previous* *round* measures the payoff received by the reference player in the previous round. *Mean* *payoff* *of* *neighbors* *in* *previous* *round* measures the average payoff received by the neighbors’ player in the previous round. The demographic characteristics includes gender (1= male, 0=female), number of neighbors, number of siblings, the field of the bachelor (1=humanities, 0=science), attending to a private or semi-private school, and attending to an urban school.

**Figure 2:**
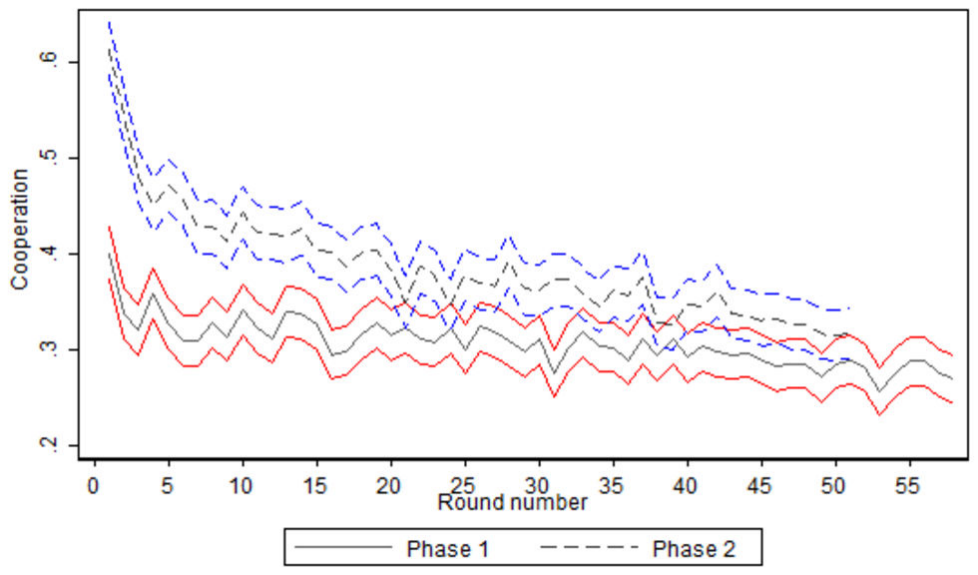
Mean cooperation, by round. [*Notes*: cooperation is defined as the mean value of a dummy variable that takes value “1” if the individual cooperates in the round of reference, and takes value “0· for defection. “Experiment” was played in the first phase of the experiment; “Control” was played in the second phase of the experiment. Round number goes from 1 to 58.].

Figure 2 shows the mean cooperation level of the sample over the rounds of the experiment, and their confidence intervals, in the 2 phases of the experiment. We analyze the 2 phases of the experiment separately because we expect to find differences in the level of cooperation between the 2 phases. The reason is that in phase 1 that was played first, reputation effects may be stronger compared to phase 2 where information on the decisions of neighbors in the previous round are shown for the previous and not the current neighbors. We observe that there is a decreasing trend in the level of cooperation in the phase 1 of the experiment, as the cooperation levels decrease from 50% in the first round to 32% in the last round of phase 1. In particular, the spearman’s rank correlation coefficient between our variable of interest and the number of rounds is -0.10, and it is statistically significant at P < 0.001 on a two-tailed test. This is consistent with the idea that reputation effects is important during the first phase of the experiment, where players decide without any previous knowledge of the underlying mechanisms of the game, while in the second phase of the experiment reputation effects disappeared as neighbors are different in each round. All this evidence is in favor of the sequential equilibrium reputation hypothesis, consistent with previous studies [28,29].

Additionally, we analyze to what extent the design of the experiment affected the behavior of players. The experiment was carried out in two phases, where in the first phase players were randomly assigned to play in a heterogeneous or lattice square network with all players having the same neighbors during this phase. For instance, for an individual playing in a heterogeneous network, this individual had the same number of neighbors, and neighbors were always the same (e.g., same identities) during all the rounds of this phase of the experiment (e.g., static network). Later, in the second phase of the experiment, the same individuals played in the same type of network (e.g., heterogeneous or square lattice) but now neighbors were different in each round (e.g., dynamic network). If we find substantial differences in the level of cooperation between the two phases, such differences may be attributed to differences in the behavior of individuals due to the type of network (e.g., static versus dynamic), given that the same individuals were assigned to the same network (e.g., heterogeneous or square lattice) in the two phases. We do not consider differences in the behavior of individuals between the heterogeneous and the square lattice network, as previous evidence has found no evidence of behavior depending of the type of network [30,31]. Such differences may be due to the fact that individuals may have learned how to play during the first phase, approaching the Nash-Equilibrium solution in the second phase.

Comparing the level of cooperation between the two phases, the mean cooperation level in phase 1 is 0.386 with a standard deviation of 0.487 (n=62679), while the mean cooperation level in the control phase is 0.307 with a standard deviation of 0.461 (n=71282). A t-type test of means of the variable for the two subsamples indicates that the difference is statistically significant at the 99% level (p<0.0001), and thus a raw comparison of the data indicates that the cooperation level is slightly lower in the second phase compared to the first one. Nevertheless, such a difference in cooperation could be explained by the “reputation hypothesis” and disappears over time as the reputation effect disappears. Thus, we next analyze how differences in the level of cooperation by phase evolved during the experiment. Figure 2 shows the mean level of cooperation over the experiment, by phase, and the confidence intervals of the mean level of cooperation. We first observe that the overall cooperation in phase 1 is larger than the overall cooperation in phase 2 in all the rounds of the experiment. However, looking at the confidence intervals for each phase, the negative confidence interval for the observations of phase 1 (${\text{x̄}}_{1}-1.96SE_{1}$) does not overlap with the positive confidence interval for observations of phase 2 (${\text{x̄}}_{2}-1.96SE_{2}$) in the first 20 rounds, but afterwards level of cooperation is similar in the two phases of the experiment (${\text{x̄}}_{1}$and *SE*_1_ measures the average cooperation and the standard error in cooperation in each round of phase 1 of the experiment. ${\text{x̄}}_{2}$and *SE*_2_ measures the average cooperation and the standard error in cooperation in each round of phase 2 the experiment). Thus, we find evidence that the gap in cooperation decreases over time, reaching similar levels of cooperation later in the game. This may indicate that the reputation hypothesis played a role in shaping individuals’ behavior in the first phase of the experiment, disappearing at later stages. Also, this evidence suggests that the two phases of the experiment must be analyzed separately.

Considering the overall earnings obtained by students, in each round the mean earning is 10.536, and the mean number of neighbors is 3.574. We also consider the earnings obtained in previous rounds for both participants and the participant’s neighbors. Since this information is shown to players in each round, we use this information as a proxy to study the effect of previous actions on the behavior of individuals. In particular, we compute the earnings obtained by the participant in the previous round, and the overall earnings obtained by the participant’s neighbors in the previous round. 

For the payoffs obtained by respondent’s neighbors in the previous round, here we must take into account that while the number of neighbors is fixed in the square lattice network (e.g., four), it varies between 2 and 16 in the heterogeneous network, and thus we need to summarize neighbors’ earnings in a single variable to avoid losing observations. Consider students who have 16 neighbors, compared to students who have less than 16 neighbors. If we want to introduce variables for the earnings of each neighbor, we will need 16 variables for students who have 16 neighbors. For students who have less than 16 neighbors, some of these 16 variables would contain no values, and thus these students would be dropped out from estimations as they would include missing values in the neighbors’ variables. We cannot compute the information of the previous round in round 1, as it is the beginning of each phase and there is no previous round, so we will exclude round 1 of each phase during the empirical analysis. In particular, for neighbors’ earnings in the previous round we have done the sum of the variable of earnings for all the neighbors in the previous round (e.g., what current neighbor played in the previous round) and then divided the sum by the number of neighbors, which yields an average value of the neighbors’ earnings in the previous round. Table 1 shows the average values of the earnings obtained by participants and the mean earnings of neighbors in previous rounds, and we observe that overall earnings obtained in the previous round and mean overall earnings of neighbors are 10.65 and 12.75 ECUs.

**Figure 3:**
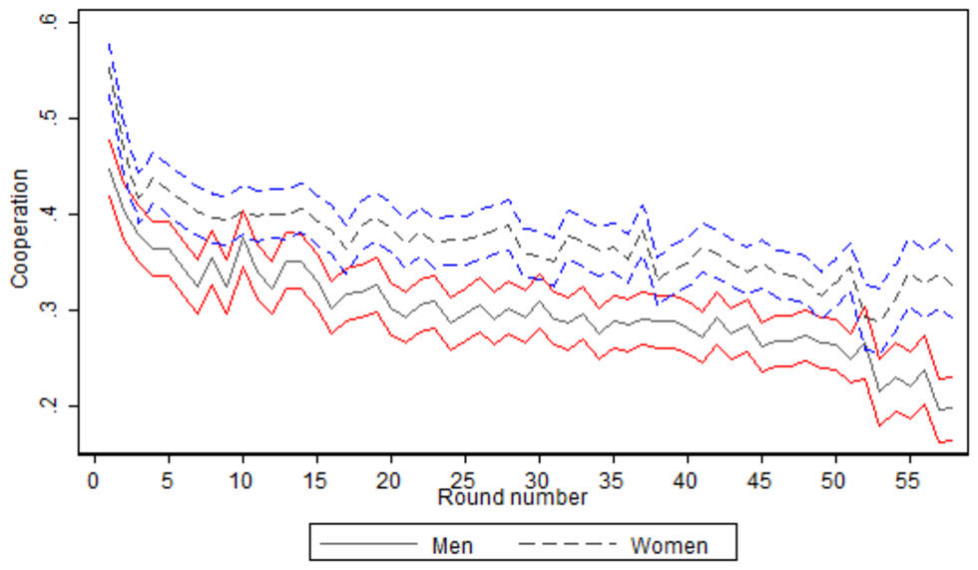
Mean cooperation, by round and gender. [*Notes*: cooperation is defined as the mean value of a dummy variable that takes value “1” if the individual cooperates in the round of reference, and takes value “0· for defection. Round number goes from 1 to 58. Confidence intervals (CI) are defined at the 95% level. Red lines show the CI for men, and blue lines show the CI for women.].

Considering the raw gender difference in cooperation, the average cooperation level of female players during the experiment is 0.375 with an standard deviation of 0.484 (n=74992), while the average cooperation level for male players is 0.304 with an standard deviation of 0.460, and a t-type test of means of the variable for the 2 subsamples indicates that the difference is statistically significant at the 99% level (p<0.0001). Thus, a raw comparison of the data indicates that female students have a higher probability of cooperation compared to male students in the experiment. However, previous evidence has shown that gender differences in cooperation are present during the first rounds of a repeated experiment, but that gender differences disappear over time [24]. Thus, we next analyze how differences in the level of cooperation by gender evolved during the experiment. Figure 3 shows the mean level of cooperation over the experiment, by gender, and the confidence intervals of the mean level of cooperation. We first observe that the overall cooperation for female players is larger than the overall cooperation for male players in all the rounds of the experiment. Additionally, looking at the confidence intervals for each gender, in most cases the negative confidence interval for female players (${\text{x̄}}_{f}-1.96SE_{f}$) does not overlap with the positive confidence interval for male players (${\text{x̄}}_{m}-1.96SE_{m}$), indicating that there is a statistically significant gender gap in cooperation throughout the different rounds of the experiment (${\text{x̄}}_{f}$and *SE*_*f*_ measures the average cooperation and the standard error in cooperation for female students in each round of the experiment. ${\text{x̄}}_{m}$and *SE*_*m*_ measures the average cooperation and the standard error in cooperation for male students in each round of the experiment). Thus, we find evidence that the gender gap in cooperation favoring female students does not disappear over time.

## Empirical Strategy

As a first specification, we estimate Ordinary Least Squares (OLS) regressions on the decision to cooperate or to defect. We estimate the following equation:

$${\text{C}}_{\text{ijt}}{\text{=α+βX}}_{i}\text{+}\delta {\text{Game}}_{ijt}{\text{+ε}}_{\text{ijt}}$$ (1)

where *C*_*ijt*_ represents the decision (cooperation/defection) by participant “i” in network “j” at round “t”. The dependent variable is a dummy variable that takes value 1 if individual “i” in network “j” at round “t” decided to cooperate, and takes value 0 if he/she decided to defect. The vector *X*_*i*_ includes participant’s “i” demographic characteristics such as gender (1= male, 0=female), number of siblings, the field of the bachelor (1=humanities, 0=science), whether the student attended a private (1=yes, o=no) or semi-private (1=yes, 0=no), and whether the high school is located in an urban area (1=yes, 0=no) or not. *Game*_ijt_ includes game variables. *ε*_*ijt*_ is a random variable (e..g., standard errors) that represents unmeasured factors, capturing all the factors that may affect participant’s decisions and for what we do not have information, and we assume thatε_ijt_~*N*(0,*σ* ^2^). We cluster observations by individual to allow for differences in the standard errors due to arbitrary intra-individual correlation. 

We have estimated Equation (1) using 6 different samples: 1) individuals playing in the heterogeneous network in phase 1, 2) individuals playing in the square lattice network in phase 1, 3) individuals playing in the heterogeneous and the square lattice networks during in phase 1, 4) individuals playing in the heterogeneous network in phase 2, 5) individuals playing in the square lattice network in phase 2, and 6) individuals playing in the heterogeneous and the square lattice networks during the phase The reason to estimate with different samples is to see if results are consistent to sample selection and network selection issues. If we obtain different results in different subsamples, it could be that networks may have effects on the decisions process of individuals, or because individuals selected into the different networks are different.

*Game*_*ijt*_ includes the following variables: the round number (j=1,2...51), the number of neighbors participant “i” is playing with, participant’s earning in the previous round, and the mean earning of neighbors in the previous round. Given that we have an iterative game, and students are shown in each round the earnings and the decision their current neighbors played in the previous round (see Figures S1, S2 and S3 in Material S1 for an example of the screen students are shown during the game), we include these variables to see whether or not participant and neighbors’ decisions in previous rounds affect the behavior of players.

We also estimate models that take into account the unobserved heterogeneity of individuals, since there may be some unobserved factors at the individual level that may be correlated with the cooperation decision, and thus results based on Equation (1) may be biased. For instance, past personal experiences, mood in the day of the experiment, or personal attitudes towards justice, equity and confidence may condition the decisions of individuals of our experiment, and if we do not take into account such differences the coefficient β in Equation (1) would be capturing the effects of such unobserved differences and not the real of factors such as gender. Thus, we estimate a random-effects linear probability model to control for unobserved heterogeneity of individuals (since we are interested on how the level of cooperation depends on gender and the number of neighbors, among others, we cannot use a fixed-effects estimator since these variables would be eliminated from estimates, and hence the random-effect estimator is preferable for our purpose), where we use the following equation:

$${\text{C}}_{\text{ijt}}{\text{=α}}_{i}{\text{+βX}}_{i}\text{+}\delta {\text{Game}}_{ijt}{\text{+ε}}_{\text{ijt}}$$ (2)

Here *C*_*ijt*_ represents the decision (cooperation/defection) by individual “i” in network “j” at round “t”, and α_*i*_represent the individual effect. The time variation needed to estimate a panel data model is given by the fact that respondents played more than one round during each phase.

The fact that the data allows to control for the unobserved heterogeneity of individuals makes the linear probability model particularly attractive with respect to other models such as the Probit model. Although the linear probability model may not provide a very good estimate of the partial effects at extreme values of the independent variables, it still produces a consistent and even unbiased estimator of the partial effects on the response probability averaged across the distribution of the independent variable. We have also estimated Probit models on the same subsamples, and results are consistent to the OLS results. See “S2. Probit results” in Material S1 for a description of the model and the estimation results.

## Results

Columns (1) to (6) in Table 2 show the results of estimating Equation (1) for the six subsamples mentioned above. Considering the effect of gender on the level of cooperation of individuals, we observe negative and statistically significant associations between being male and the probability of cooperation in all the analyzed samples, with these associations being statistically significant at standard levels. In particular, we find that being male is associated with a decrease of between 4 (Column (1)) and 8 (Column (4)) percentage points in the probability of cooperation. Considering that the mean level of cooperation during the experiment is 0.341, we find that female players have a higher probability of between 11 and 23 percentage points to cooperate, compared to male players. This negative association between being male and cooperation is present when we analyze alternative samples and networks (e.g., phase 1 versus phase 2), and is net out of the effects of other demographic characteristics. For instance, it could be that the number of siblings of the respondent conditions his/her cooperative behavior, fostering the level of cooperation of the individuals as he/she is more used to intra-household bargaining. Also, it could be that children who attend a private school observe a higher household income, which may make those children to give a relative lower value to the payoffs offered during the game, affecting their behavior. These and more factors may be driving our results, and thus we need to control for these characteristics in our estimations to net out the effect of gender from other effects.

**Table 2:** OLS Regressions for cooperation of individuals playing the Prisoner’s Dilemma.

**OLS Regressions**	**(1)**	**(2)**	**(3)**	**(4)**	**(5)**	**(6)**
	***Heterogenous’s Experiment***	***Sq. Lattice Experiment***	***Het + Sq Lattice Experiment***	***Heterogenous Control***	***Sq. Lattice Control***	***Het + Sq Lattice Control***
***Male***	-0.038***	-0.061***	-0.050***	-0.081***	-0.068***	-0.073***
	(0.013)	(0.012)	(0.009)	(0.016)	(0.016)	(0.012)
***Round number***	-0.003***	-0.003***	-0.003***	-0.001***	-0.001***	-0.001***
	(0.000)	(0.000)	(0.000)	(0.000)	(0.000)	(0.000)
***Number of neighbors***	-0.004	-	-0.004	-0.001	-	-0.002
	(0.004)	-	(0.003)	(0.004)	-	(0.004)
***Humanities***	0.012	0.037***	0.024**	0.020	0.028	0.025**
	(0.014)	(0.013)	(0.010)	(0.018)	(0.017)	(0.013)
***Number of siblings***	0.004	0.000	0.002	-0.012	0.009	0.000
	(0.007)	(0.006)	(0.005)	(0.009)	(0.008)	(0.006)
***Attending a private school***	0.017	-0.040**	-0.017	0.041	-0.027	0.000
	(0.022)	(0.020)	(0.016)	(0.033)	(0.026)	(0.021)
***Attending a Semi-private school***	-0.010	-0.016	-0.013	0.010	-0.024	-0.008
	(0.014)	(0.014)	(0.010)	(0.019)	(0.019)	(0.013)
***Attending an urban school***	-0.001	-0.010	-0.006	-0.032*	-0.010	-0.022
	(0.014)	(0.015)	(0.010)	(0.018)	(0.020)	(0.014)
***Mean payoff of neighbors in previous round***	0.004***	0.009***	0.005***	0.000	0.001	0.000
	(0.001)	(0.001)	(0.000)	(0.000)	(0.001)	(0.000)
***Payoff in previous round***	-0.003***	-0.004***	-0.004***	-0.007***	-0.008***	-0.007***
	(0.000)	(0.000)	(0.000)	(0.000)	(0.000)	(0.000)
***Constant***	0.475***	0.395***	0.458***	0.480***	0.432***	0.462***
	(0.021)	(0.021)	(0.017)	(0.023)	(0.024)	(0.020)
**Observations**	30200	31250	61450	34428	35625	70053
**Number of id**	604	625	1229	625	604	1229
**R-squared**	0.03	0.04	0.03	0.03	0.03	0.03

Note: Sample consists of final-year high school students from Aragon (Spain). Clustered standard errors in parenthesis. We estimate the following equation: C_ijt_=α+βX_*i*_+*δ*Game_*ijt*_+ε_ijt_where *C*_*ijt*_ represents the decision (cooperation/defection) by individual “i” in network “j” at round “t”. The dependent variables is a dummy variable that takes value 1 if individual “i” in network “j” at round “t” decided to cooperate, and takes value 0 if he/she decided to defect. The vector *Xi* includes participant’s “i” demographic characteristics such as gender (1= male, 0=female), number of siblings, the field of the bachelor (1=humanities, 0=science), while *Game*_*ijt*_ includes game variables from the previous round. We cluster observations by individual to allow for differences in the variance/standard errors due to arbitrary intra-individual correlation. *** Significant at the 1% level; ** Significant at the 5% level; * Significant at the 10% level.

Other factors affecting the level of cooperation of individuals are the round number, the respondent’s payoff in the previous round, and the mean payoff of neighbors in the previous round. In the case of the round number, we observe a negative and statistically significant association between the round number and the level of cooperation in both phase 1 (Columns (1) to (3)) and phase 2 of the experiment (Columns (4) to (6)). In particular, we find that an additional round in the experiment decreases the probability of cooperation by 0.3 and 0.1 percentage points in phase 1 and 2 of the experiment. These results are consistent with the “sequential equilibrium reputation hypothesis” since the cooperation level decreases as the game unravels to mutual defection. However, we observe that the effect of the round in the level of cooperation is lower in the phase 2 of the experiment, which may indicate that the reputation hypothesis has a smaller effect in phase 2 of the experiment where players may have learned that reputation is not an underlying mechanism operating during the game, or they may have realized that as their neighbors change in every round the idea of reputation does not apply.

Respondent’s payoff in the previous round has a negative and statistically significant association with the probability of cooperation. In particular, each additional ECU obtained in the previous round is related with decreases in the probability of cooperation of around 0.4 percentage points in phase 1 of the experiment, and of around 0.07 percentage points in phase 2 of the experiment. One explanation for this reported association is that if respondent has defected in the previous round, and he/she has obtained a high payoff in the previous round, the respondent is in a “defection mood” to try to obtain a high payoff in the current round. The opposite applies for the mean payoffs obtained by neighbors in the previous round, as it has a positive and statistically significant association with the level of cooperation. Furthermore, this positive association is not present in the phase 2 of the experiment, where neighbors are different in each round. Our experimental findings suggest that the behavior of the players depends on the previous actions of their neighbors (conditional cooperation), but may also depend on the previous action of the players themselves (moody conditional cooperation), indicating that players seem to react to the context in a way influenced by their own previous action [32,33]. However, it is only for static networks where the “moody conditional cooperation” is present, indicating that in networks where neighbors change during the process the behavior of players does not depend on their neighbors. This, in fact, reinforces our interpretation that players realize the difference between the two treatments and act accordingly. 

Previous research on gender differences in economic experiments has shown that there are gender differences in risk, social and competitive preferences (see 25 for a review). Additionally, there can be other socio-demographic factors not controlled for in the experiment, such as the respondent’s household income, that can be correlated with the higher level cooperation of women. Thus, the previously observed gender difference in cooperation could be attributed to gender differences in preferences, or to non-controlled socio-demographic factors. For this reason, we need to apply an econometric technique that nets out the effect of gender from other observed (although not controlled for) and unobserved factors (e.g., preferences). If we now consider that our dataset has a panel data structure (e.g., same individuals observed during several periods of time), we can apply the Random Effect estimator [34] to estimate the effect of gender on cooperation net out of observed and unobserved heterogeneity. 

Columns (1) to (6) in Table 3 show the results of estimating Equation (2) for the six subsamples we are working with. We observe negative and statistically significant associations between being male and the probability of cooperation in all the analyzed samples, with these associations being statistically significant at standard levels. In particular, we find that being male is associated with a decrease of between 4 (Column (1)) and 8 (Column (4)) percentage points in the probability of cooperation. For the rest of factors, our results are consistent compared to the OLS results, and indicate that our observations cannot be attributed to non-controlled factors. 

**Table 3:** Random effects regressions for cooperation of individuals playing the Prisoner’s Dilemma.

**RE Regressions**	**(1)**	**(2)**	**(3)**	**(4)**	**(5)**	**(6)**
	***Heterogenous Experiment***	***Sq. Lattice Experiment***	***Het + Sq Lattice Experiment***	***Heterogenous Control***	***Sq. Lattice Control***	***Het + Sq Lattice Control***
***Male***	-0.038***	-0.063***	-0.051***	-0.082***	-0.069***	-0.074***
	(0.012)	(0.010)	(0.009)	(0.014)	(0.013)	(0.010)
***Round number***	-0.003***	-0.003***	-0.003***	-0.001***	-0.001***	-0.001***
	(0.000)	(0.000)	(0.000)	(0.000)	(0.000)	(0.000)
***Number of neighbors***	-0.008**	-	-0.007**	-0.005	-	-0.007**
	(0.003)	-	(0.003)	(0.004)	-	(0.003)
***Humanities***	0.011	0.038***	0.025**	0.020	0.028*	0.026**
	(0.014)	(0.012)	(0.010)	(0.017)	(0.015)	(0.011)
***Number of Brothers***	0.004	0.001	0.002	-0.012	0.009	0.001
	(0.008)	(0.006)	(0.005)	(0.009)	(0.007)	(0.006)
***Attending a private school***	0.018	-0.040**	-0.018	0.040	-0.028	-0.001
	(0.027)	(0.019)	(0.017)	(0.032)	(0.024)	(0.020)
***Attending a Semi-private school***	-0.010	-0.015	-0.012	0.010	-0.024	-0.008
	(0.014)	(0.012)	(0.010)	(0.016)	(0.015)	(0.011)
***Attending an urban school***	-0.001	-0.010	-0.006	-0.032**	-0.010	-0.022**
	(0.014)	(0.013)	(0.010)	(0.016)	(0.016)	(0.011)
***Mean payoff of neighbors in previous round***	0.004***	0.005***	0.005***	0.000	0.001**	0.000
	(0.000)	(0.000)	(0.000)	(0.000)	(0.001)	(0.000)
***Payoff in previous round***	-0.002***	-0.003***	-0.003***	-0.006***	-0.006***	-0.006***
	(0.000)	(0.000)	(0.000)	(0.000)	(0.000)	(0.000)
***Constant***	0.467***	0.445***	0.472***	0.478***	0.408***	0.461***
	(0.020)	(0.018)	(0.016)	(0.022)	(0.020)	(0.018)
**Observations**	30200	31250	61450	34428	35625	70053
**Number of id**	604	625	1229	625	604	1229
**R-squared**	0.03	0.04	0.03	0.03	0.03	0.03

Note: Sample consists of final-year high school students from Aragon (Spain). Standard errors in parenthesis. We estimate the following equation: C_ijt_=α_*i*_+βX_*i*_+*δ*Game_*ijt*_+ε_ijt_where *C*_*ijt*_ represents the decision (cooperation/defection) by individual “i” in network “j” at round “t”. The dependent variables is a dummy variable that takes value 1 if individual “i” in network “j” at round “t” decided to cooperate, and takes value 0 if he/she decided to defect. The vector *Xi* includes participant’s “i” demographic characteristics such as gender (1= male, 0=female), number of siblings and the field of the bachelor (1=humanities, 0=science), while *Game*_*ijt*_ includes game variables from the previous round. *** Significant at the 1% level; ** Significant at the 5% level; * Significant at the 10% level.

## Conclusions

The question about whether or not cooperation of individuals varies systematically with the sex of the decision maker is analyzed in this paper. Prior research on the prisoner’s dilemma, social dilemmas, and public goods provision has found mixed results from a gender perspective. We contribute to the literature by analyzing gender differences in cooperation for Spanish high school students. To that end, we reanalyzed data from an experiment with 1,229 volunteers from final-year high-school students (17-18 years old) of 42 high schools located in the Region of Aragón. Standard econometric techniques used in the field of economics (i.e., Ordinary Least Squares, and Random Effects models) are applied to analyze gender differences in the level of cooperation of Aragonians high school students.

We find that being male is negatively associated with the level of cooperation, with this association being statistically significant at standard levels. We also obtain a gender difference in the level of cooperation when we control for the unobserved heterogeneity of individuals, which indicates that the gender gap in cooperation favoring female students is present after netting out this effect from other socio-demographics factors not controlled for in the experiment, and from gender differences in risk, social and competitive preferences (see 25 for a review). Thus, our results point toward a gender difference in the level of cooperation that might be attributed to a genetic factor. 

We hope that this article will serve as a resource for those in the field of economics seeking to understand gender differences in the level of cooperation, and to use it as a starting point to illuminate the debate on genetic differences in behavior. We urge researchers to routinely record the gender of the participants when possible in order to expand our understanding of gender differences. Having established this gender difference, it is appropriate now for research to address the issue of how other parameters of the experimental setting influence the behavior of women and men. For instance, gender may affect how individuals punish non-cooperative partners, one of the most relevant mechanisms to foster cooperation [35-37]. In the same spirit, when players can choose their partners by rewiring their connections [38-40] gender may also be responsible for different behaviors. Finally, to the extent that our findings support the existence of gender differences in cooperation, the theory used to model some economic outcomes, such as household bargaining, could incorporate this gender asymmetry. We leave all these issues for future research.

## Supporting Information

Material S1 (DOCX)
