# Supplementary material for: Gender Differences in Cooperation: Experimental Evidence on High School Students
Source: PLoS One. 2013 Dec 18;8(12):e83700. doi: 10.1371/journal.pone.0083700 (PMC3867463; doi:10.1371/journal.pone.0083700)
Supplement: Material S1 — (DOCX) [file pone.0083700.s001.docx]

**Supplementary Material for**

**Gender differences in cooperation: experimental evidence on high school students**[[1]](#footnote-1)

**José Alberto Molina^1,2^, J. Ignacio Giménez-Nadal^1^, José A. Cuesta^3,4^, Carlos Gracia-Lazaro^3^, Yamir Moreno^3,5^ and Angel Sanchez^3,4^**

**S1. The Experiment**

The experiment was run using a web application specifically developed to this purpose, using free software. It was developed in Ruby On Rails, and has a MySQL database, a freely available open source relational database management system based on Structured Query Language (SQL), the most popular language for adding, accessing and managing content in a database.

The application was designed to be used by three different user profiles. First of all, we have all the players. Secondly, there are teachers who were responsible for supervising students through their dedicated web monitors, facilitating the work of the central administrator work and contributing to the success of the experiment. Finally, the administrators were responsible for controlling the game and everything that was happening in real time. There was a last participant, a daemon or process running in the background whose function was to update the results and play instead of players who do not play within the specified time frame for each action (20 seconds). Considering that the experiment required that around 1300 students could play online simultaneously, we used a server with enough power, and many optimizations were performed in terms of connections to the server, access to database, client-server data exchange, lightness of the interface, control logic, etc.

***On-line tutorial for players***

The following is a translation of the Spanish original on-line tutorial (available upon request). It is worth remarking that each player had a customized pair of colors and a corresponding number of neighbors. We refer in what follows to the latter as X (but X showed its actual value for each participant). As advanced above, to avoid framing effects, the two actions were always referred to in terms of colors (chosen randomly among a predefined set of possible pairs of colors), and the game was never referred to as Prisoner’s Dilemma in the material handed to the volunteers. Subjects were properly informed of the consequences of choosing each action, and some examples were given to them in the introduction. After every round subjects were given the information of the actions taken by their neighbors and their corresponding earnings. In all cases, the earnings were properly normalized to avoid the possibility of guessing the number of connections of their neighbors. The instructions are given here assuming a given pair of colors (green and brown), but again, each participant saw the actual color assigned to him/her. Moreover, we took into consideration the possibility that some of the students were colorblind. In this sense, we provided clear instructions to avoid any possible error in the final results, specifying the order in which each color appeared on the screen and also using a combination of specifically selected colors (5 different pairs) so that the probability of error was reduced to a minimum.

**Page 1**

This is an experiment designed to study how individuals make decisions.

You are not expected to behave in any particular way.

Whatever you do will determine the amount of money you can earn.

You have a written version of these directions which you can check at any stage of the experiment.

Please keep quiet during the experiment. If you need help, raise your hand and wait to be attended.

**Page 2: Directions to participate in the experiment**

This experiment consists of TWO (2) parts.

Each part consists of an undetermined number of ROUNDS (approximately between 50 and 70, but there might be more or less).

Each part will last at most 35 minutes, but could finish before.

In each part you will be able to earn different amounts of money, depending on the decisions that you and the rest of participants make in every round.

The earning of each round is given in a monetary unit called ECU. When the experiment finishes, an exchange rate from ECUs to Euros will be established as a function of the number of participants.

Your total earning in this experiment will be the accumulated earnings in all the rounds of the two parts, plus a show-up fee.

**Page 3: A Round**

In each ROUND you will be placed in a node of a virtual NETWORK.

In this network you will be linked to X (*here the actual number is shown to each participant*) people, whom we shall refer to as “neighbors”.

Your neighbors will also be connected to other people. You will be one of those neighbors, but the rest of them will not necessarily be the same neighbors that you have.

You will never know who your neighbors are, and nobody will know if you are her neighbor either.

The network is virtual. People around you in the room are not necessarily your neighbors.

**Page 4: Decision to make in every round**

Every round, each of the participants must choose a color: GREEN or BROWN. (*Note: as explained before, each participant sees the actual colors chosen for them. For clarity, we henceforth refer to green and brown*)

To choose a color you just have to click a button appearing in the screen.

Each time you choose a color (either blue or yellow) you will earn an amount of money which will depend on yours and your X neighbors’ choices.

If you choose GREEN and your neighbor also chooses GREEN, each receives 7 ECUs.

If you choose GREEN and your neighbor chooses BROWN, you receive 0 ECUs and your neighbor 10 ECUs.

If you choose BROWN and your neighbor also chooses BROWN, each receives 0 ECUs.

If you choose BROWN and your neighbor chooses GREEN, you receive 10 ECUs and your neighbor 0 ECUs.

These rules are the same for all participants.

**Page 5: Possible earnings per neighbor**

In the following table each row corresponds to the decision you can make and each column correspond to one of your neighbors’ decision.

**
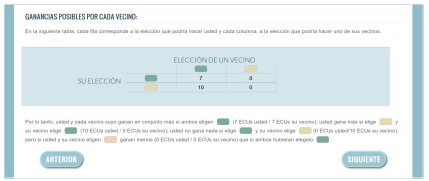
**

**Figure S1. Snapshot of the experimental software.**

Consider that:

• you and each of your neighbors will globally earn more if you both choose GREEN (7 ECUs you / 7 ECUs your neighbor);

• you will earn more if you choose BROWN and your neighbor chooses GREEN (10 ECUs you / 0 ECUs your neighbor);

• but if both you and your neighbor choose BROWN you both will earn less (0 ECUs you / 0 ECUs your neighbor) than if you both chose GREEN.

**
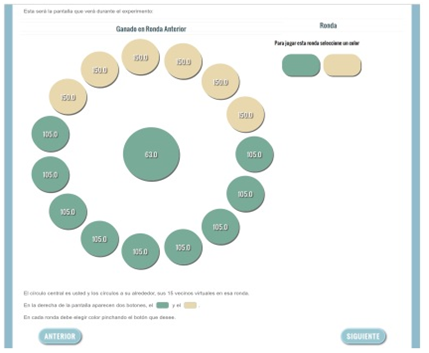
**

**Figure S2. Snapshot of the experimental software. [***Note*: earnings shown do not correspond to any real situation, but simply illustrate how they were seen by the subjects.]

This is the screen you will be seeing during the experiment. The central circle represents you, and the surrounding circles represent your virtual neighbors in that round.

On the right of the screen you will see two buttons: GREEN and BROWN.

Each round you must choose one of them clicking the corresponding button.

**Page 7: These are some examples of what you could earn in a round:**

Example 1: Imagine you choose GREEN, 3 of your neighbors choose GREEN and 1 chooses BROWN. In that round you will earn 3X7+1X0=21 ECUs.

Example 2: In another round you choose BROWN, 2 of your neighbors choose GREEN and 2 choose BROWN. In that round you will earn 2X10+2X0=20 ECUs.

**Page 8: Round iteration**

Remember that each part will consist of an undetermined number of rounds.

Each round you will have up to 20 seconds to choose a color. After these 20 seconds, if you didn’t choose, the system will choose for you. Whatever happens it will not affect the behavior of the system in the next rounds: you will be able to make your subsequent choices normally. (Don’t worry: 20 seconds are more than enough to make a choice).

The round will not end until all participants have made their choice.

At the end of each round you will see a screen like this one:

**
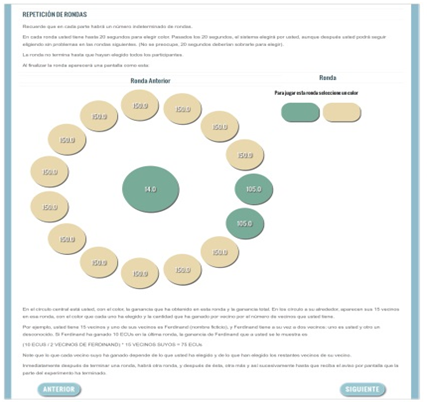
**

**Figure S3: Snapshot of the experimental software. [***Note*: Earnings shown do not correspond to any real situation, but simply illustrate how they were seen by the subjects.]

**Page 9: Part I of the experiment**

In this part the system will randomly assign each participant to a given node of the virtual network.

**This place will be kept fixed until this part ends.**

This means that you will be interacting with **the same X neighbors** during all rounds of this part.

Remember that in each round you must choose a color.

When this part finishes, you will be notified and will see the directions for the next part.

(Part I begins.)

**Page 10: Part I of the experiment has finished.**

Please, keep quiet.

Part II will start in a few seconds.

**Page 11: Part II of the experiment**

In this part, **before each round begins,** every participant will be moved to a **new** random node of the virtual network. Therefore, in general **you will likely have X new neighbors every round.**

This means that **the node you are in will be changing along the experiment.**

Thus you will NOT be linked all rounds to the same X neighbors.

**Page 12: The rules to make decisions every round are the same as in Part I.**

The only thing that is different is that your neighbors will most likely not be the same every round.

Remember:

• Every round you have 20 seconds to make a choice.

• The round finishes only when all participants have made their decisions.

• At the end of each round you will be seeing a screen like in Part I

(Part II begins.)

Page 13: Part II of the experiment has finished.

Please, keep quiet.

The experiment has not finished yet.

You have to answer the following questionnaire.

Please, answer ALL questions in the questionnaire that you will be shown immediately.

(*The questionnaire was shown and afterwards they were notified how much they had earned and were to go to get paid.*)

*Synchronous play and automatic actions*

The experiment assumes synchronous play, thus we had to make sure that every round ended in a certain amount of time. This playing time was set to 20 seconds, which was checked during the testing phase of the programs to be enough to make a decision, while at the same time not too long to make the experiment boring to fast players. If a player did not choose an action within these 20 seconds, the computer made the decision instead. This automatic decision was randomly chosen to be the player’s previous action 90% of the times and the opposite action 10% of the times. Note that we did not inform the volunteers of this procedure, only that the computer would play by them if they did not play in time. We preferred not to let them know the precise intervention to avoid players using this fact to play the same action instead of explicitly choosing an action. In any case, for the reliability of the experiment it is important that a huge majority of actions were actually played by humans, not by the computer. This quantity, when averaged over all rounds, yields that the 90% of the actions were chosen by humans, regardless of the underlying network of contacts.

**S2. Probit results**

We have alternatively estimated Probit models that take into account the dichotomous behavior of our dependent variable, in order to see the consistency of our results to alternative specifications of the econometric model. The Probit model can be estimated as follows:

$\text{Y}_{_{\text{ijt}}}^{*}\text{=βX}_{i}\text{+}\delta\text{Game}_{ijt}\text{+ε}_{\text{ijt}}$ (S1)

where $\text{Y}_{_{\text{ijt}}}^{*}$is a latent variable, and $\text{ε}_{\text{ijt}}\sim N(0,1)$. Then $\text{Y}_{ijt}$can be viewed as an indicator for whether this latent variable is positive:

$\text{C}_{ijt}=\left\{\begin{matrix} 1\text{ if Y}_{_{ijt}}^{*}>0\text{ i.e. -}\varepsilon_{ijt}<\text{βX}_{i}\text{+}\delta\text{Game}_{ijt} \\ 0\text{ otherwise} \end{matrix}\right.$ (S2)

The use of the standard normal distribution causes no loss of generality compared with using an arbitrary mean and standard deviation because adding a fixed amount to the mean can be compensated by subtracting the same amount from the intercept, and multiplying the standard deviation by a fixed amount can be compensated by multiplying the weights by the same amount. Table S1 shows the results of estimating Probit models on the 6 subsamples used throughout the paper.

**Table S1. Probit regressions for cooperation of individuals playing the Prisoner Dilemma.**

| **Probit Regressions** | **(1)** | **(2)** | **(3)** | **(4)** | **(5)** | **(6)** |
| --- | --- | --- | --- | --- | --- | --- |
|  | ***Heterogeneous Experiment*** | ***Sq. Lattice Experiment*** | ***Het + Sq Lattice Experiment*** | ***Heterogeneous Control*** | ***Sq. Lattice Control*** | ***Het + Sq Lattice Control*** |
| ***Male*** | -0.102*** | -0.168*** | -0.136*** | -0.236*** | -0.206*** | -0.217*** |
|  | (0.033) | (0.034) | (0.024) | (0.048) | (0.051) | (0.035) |
| ***Round number*** | -0.007*** | -0.007*** | -0.008*** | -0.003*** | -0.004*** | -0.004*** |
|  | (0.001) | (0.001) | (0.000) | (0.001) | (0.001) | (0.000) |
| ***Number of neighbors*** | -0.013 | - | -0.011 | -0.006 | - | -0.009 |
|  | (0.010) | - | (0.009) | (0.015) | - | (0.014) |
| ***Humanities*** | 0.030 | 0.103*** | 0.065** | 0.060 | 0.083* | 0.076** |
|  | (0.037) | (0.035) | (0.026) | (0.052) | (0.050) | (0.036) |
| ***Number of Brothers*** | 0.011 | 0.001 | 0.006 | -0.032 | 0.027 | 0.002 |
|  | (0.019) | (0.016) | (0.013) | (0.027) | (0.024) | (0.018) |
| ***Attending a private school*** | 0.045 | -0.113** | -0.046 | 0.116 | -0.085 | 0.000 |
|  | (0.058) | (0.057) | (0.043) | (0.093) | (0.082) | (0.062) |
| ***Attending a Semi-private school*** | -0.026 | -0.045 | -0.035 | 0.029 | -0.074 | -0.024 |
|  | (0.037) | (0.040) | (0.028) | (0.054) | (0.058) | (0.039) |
| ***Attending an urban school*** | -0.003 | -0.025 | -0.014 | -0.094* | -0.028 | -0.065* |
|  | (0.036) | (0.040) | (0.027) | (0.053) | (0.060) | (0.040) |
| ***Mean payoff of neighbors in previous round*** | 0.010*** | 0.024*** | 0.014*** | -0.001 | 0.003* | 0.000 |
|  | (0.001) | (0.002) | (0.001) | (0.001) | (0.002) | (0.001) |
| ***Payoff in previous round*** | -0.009*** | -0.010*** | -0.011*** | -0.024*** | -0.024*** | -0.024*** |
|  | (0.001) | (0.001) | (0.001) | (0.001) | (0.001) | (0.001) |
| ***Constant*** | -0.046 | -0.263*** | -0.088* | 0.009 | -0.134* | -0.034 |
|  | (0.055) | (0.057) | (0.046) | (0.069) | (0.069) | (0.062) |
|  |  |  |  |  |  |  |
| **Observations** | 30200 | 31250 | 61450 | 34428 | 35625 | 70053 |
| **Number of id** | 604 | 625 | 1229 | 625 | 604 | 1229 |
| **R-squared** | 0.02 | 0.03 | 0.03 | 0.03 | 0.03 | 0.03 |

*Note:* Sample consists of final-year high school students from Aragon (Spain). Clustered standard errors in parenthesis. We estimate the following equation: $C_{_{\text{ijt}}}^{*}\text{=βX}_{i}\text{+}\delta\text{Game}_{ijt}\text{+ε}_{\text{ijt}}$ where $C_{_{\text{ijt}}}^{*}$is a latent variable, and $\text{ε}_{\text{ijt}}\sim N(0,1)$. Then $\text{C}_{ijt}$can be viewed as an indicator for whether this latent variable is positive, where *C_ijt_* represents the decision (cooperation/defection) by individual “i” in network “j” at round “t”. The dependent variables is a dummy variable that takes value 1 if individual “i” in network “j” at round “t” decided to cooperate, and takes value 0 if he/she decided to defect. The vector Xi includes participant’s “i” demographic characteristics such as gender (1= male, 0=female), number of siblings and the field of the bachelor (1=humanities, 0=science), while *Game_ijt_* includes game variables from the previous round.

1. [↑](#footnote-ref-1)
